# EOS® is reliable to evaluate spinopelvic parameters: a validation study

**DOI:** 10.1186/s12880-023-01178-0

**Published:** 2024-02-06

**Authors:** Mohammadreza Shakeri, Seyed Mani Mahdavi, Masih Rikhtehgar, Mohammad Soleimani, Hasan Ghandhari, Behnam Jafari, Seyedehsan Daneshmand

**Affiliations:** 1 Bone and Joint Reconstruction Research Center, Department of Orthopedics, School of Medicine, Iran University of Medical Sciences, Tehran, Iran; 2 Department of Epidemiology, School of Public Health, Iran University of Medical Sciences, Tehran, Iran

**Keywords:** EOS, Sagittal Balance, Spinopelvic parameters, Validity, Reliability

## Abstract

**Background:**

Sagittal and coronal standing radiographs have been the standard imaging for assessing spinal alignment. However, their disadvantages include distortion at the image edges and low interobserver reliability in some parameters. EOS® is a low-dose biplanar digital radiographic imaging system that can avoid distortion by obtaining high-definition images.

**Methods:**

This study aimed to evaluate spinopelvic parameters in conventional lateral C1S1 upright radiographs and EOS® images and compare them. Patients with non-deformity changes were subjected to routine clinical examinations. Plain AP and lateral X-ray radiographs were obtained along the entire spine length. Patients were also referred for full-length EOS® of the spine. Thoracic Kyphosis (TK), Lumbar Lordosis (LL), Pelvic Tilt (PT), Sacral Slope (SS), Pelvic Incidence (PI), and Sagittal Vertical Axis (SVA) were measured in the two studies by an orthopedic surgeon and a radiologist using PACS software. Also, the orthopedic surgeon evaluated the studies again after two weeks. Intra- and inter-observer reliability was then assessed using the interclass correlation coefficient (ICC). Also, the coefficient of variation was used to assess intra- and inter-observer reliability. Bland-Altman plots were drawn for each parameter.

**Results:**

The mean age was 48.2 ± 6.6 years. Among the 50 patients, 30 (60%) were female. The mean ICC for TK, LL, PT, SS, PI, and SVA in EOS® images are 0.95, 0.95, 0.92, 0.90, 0.94, and 0.98, respectively, and in C1S1 radiography images, it was 0.92, 0.87, 0.94, 0.88, 0.93, and 0.98, respectively which shows good to excellent results. The coefficient of variation for intraobserver reliability was relatively low (< 18.6%), while it showed higher percentages in evaluating interobserver reliability (< 54.5%). Also, the Bland-Altman plot showed good agreement for each parameter.

**Conclusion:**

Spinopelvic parameters, e.g., TK, LL, SS, PI, and SS, in EOS® are reliable and comparable to those in conventional lateral upright C1S1 radiographs.

## Introduction

Different components of the musculoskeletal system are biomechanically related and can affect each other [1–4]. The morphology and alignment of the pelvis and spine affect each other [1–4]. Balance in maintaining body posture in the static and dynamic state is achieved when body parts are placed together in such a way that energy consumption is minimized [4–6]. In other words, maintaining the normal alignment of the spine, pelvis, and lower limbs in the sagittal plane is necessary to maintain a standing position with minimal energy consumption [7, 8].

Sagittal and coronal malalignment of the spine is associated with back pain and disability [9–11]. Also, it causes a lower quality of life and reduced activities of daily living [12]. Spinal alignment is affected by weight bearing; thus, it is common practice to measure the spinal alignment by standing whole spinal radiographs [13]. For over 70 years, the gold standard for measuring spinal alignment has been sagittal and coronal standing whole spinal radiographs [13]. However, they have some downsides, including distortion at the edges of the image and lower interobserver reliability in some parameters [13, 14].

EOS® is a low-dose biplanar digital radiographic imaging system with two linear X-ray radiographic sources and two gaseous detector arrays moving together to scan the patient [15]. By obtaining high-definition images, EOS® can avoid distortion [16]. Moreover, it can obtain 3D images in addition to 2D images [15]. Additionally, measuring pelvic and acetabular indicators has been shown to be reliable using EOS®, similar to conventional radiology. Moreover, it offers the feature of less irradiation [17]. Most importantly, this novel imaging system is able to capture a 170 cm long image with the movement of two tubes and detectors without needing to stitch multiple images [18]. It has also been shown to be highly correlated with computed tomography (CT) for hip measurements, and significantly less irradiation [18]. Further, it can be as reliable as conventional X-rays for the grading of osteoarthritis of the knee in the anteroposterior view [19]. However, high cost and large size limit its clinical use [20]. Nevertheless, it has gained popularity in the past few years.

The present study aimed to assess the reliability of conventional lateral C1S1 radiographs for measuring spine parameters compared to EOS® images in patients presenting to the spine clinic.

## Materials and methods

### Participant enrollment

We enrolled consecutive patients presenting from April 2021 to April 2022 to the spine clinic of Shafa Yahyaian Hospital, Tehran, Iran. The convenience sampling method was used. The inclusion criteria were 18–65 years of age and failed conservative treatment. The exclusion criteria were evidence of hip dysplasia, avascular necrosis of the femoral head, any orthopedic condition in the pelvis or hip joint, spinal deformity, or a history of spinal surgery. This study was approved by the Ethics Committee of Iran University of Medical Sciences (IR.IUMS.FMD.REC.1401.091), and all participants provided written informed consent before participation. Demographic data, including age and sex, were recorded. Plain anteroposterior (AP) and lateral whole spinal X-ray radiographs were obtained. Patients were also referred for whole spinal EOS®.

### Image acquisition

**Fig. 1:**
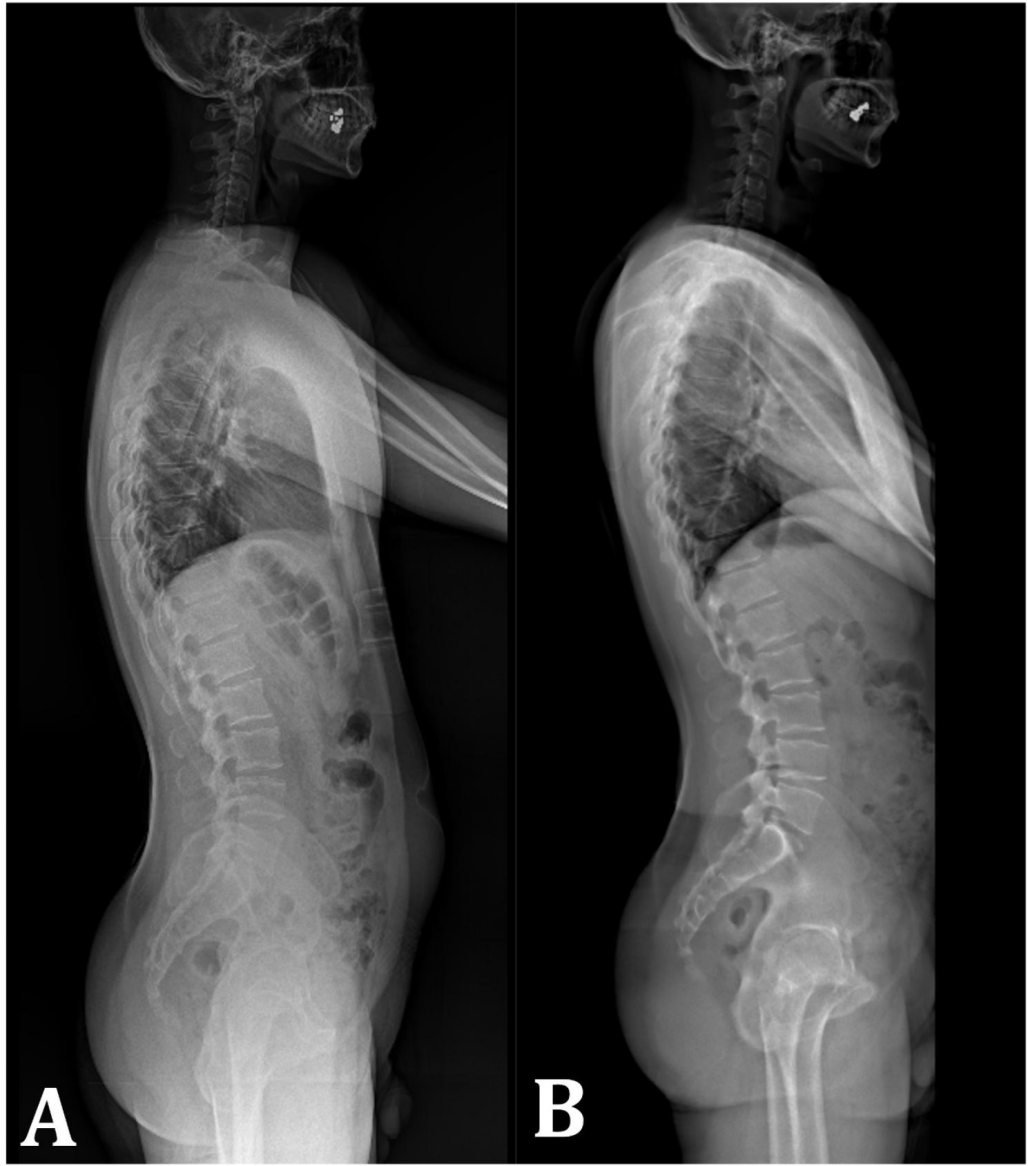
**(a)** Conventional C1S1 radiograph. **(b)** EOS^®^ image

Spinopelvic parameters, including thoracic kyphosis (TK), lumbar lordosis (LL), pelvic tilt (PT), sacral slope (SS), pelvic incidence (PI), and sagittal vertical axis (SVA), were measured in conventional C1S1 radiography and EOS®. Conventional C1S1 radiographs were obtained as standing full-length radiographs in which the heads of both femurs were visible, hip and knee joints were in full extension, elbows were flexed, and hands were placed on the contralateral clavicles. All measurements were made using PersianGulf PACS software (version 2510; Raouf Medical Group, Tehran, Iran). The spinopelvic parameters on both imaging studies were evaluated by a radiologist and an orthopedic surgeon, independently. The radiologist was an attending physician whose field of expertise was musculoskeletal radiology, and the orthopedic surgeon was an attending spine surgeon. The orthopedic surgeon reviewed the images two weeks later. Inter- and intraobserver reliability was evaluated using the interclass correlation coefficient (ICC). Figure 1 shows the conventional and EOS® imagings in one patient.

### Parameter measurement

**Fig. 2:**
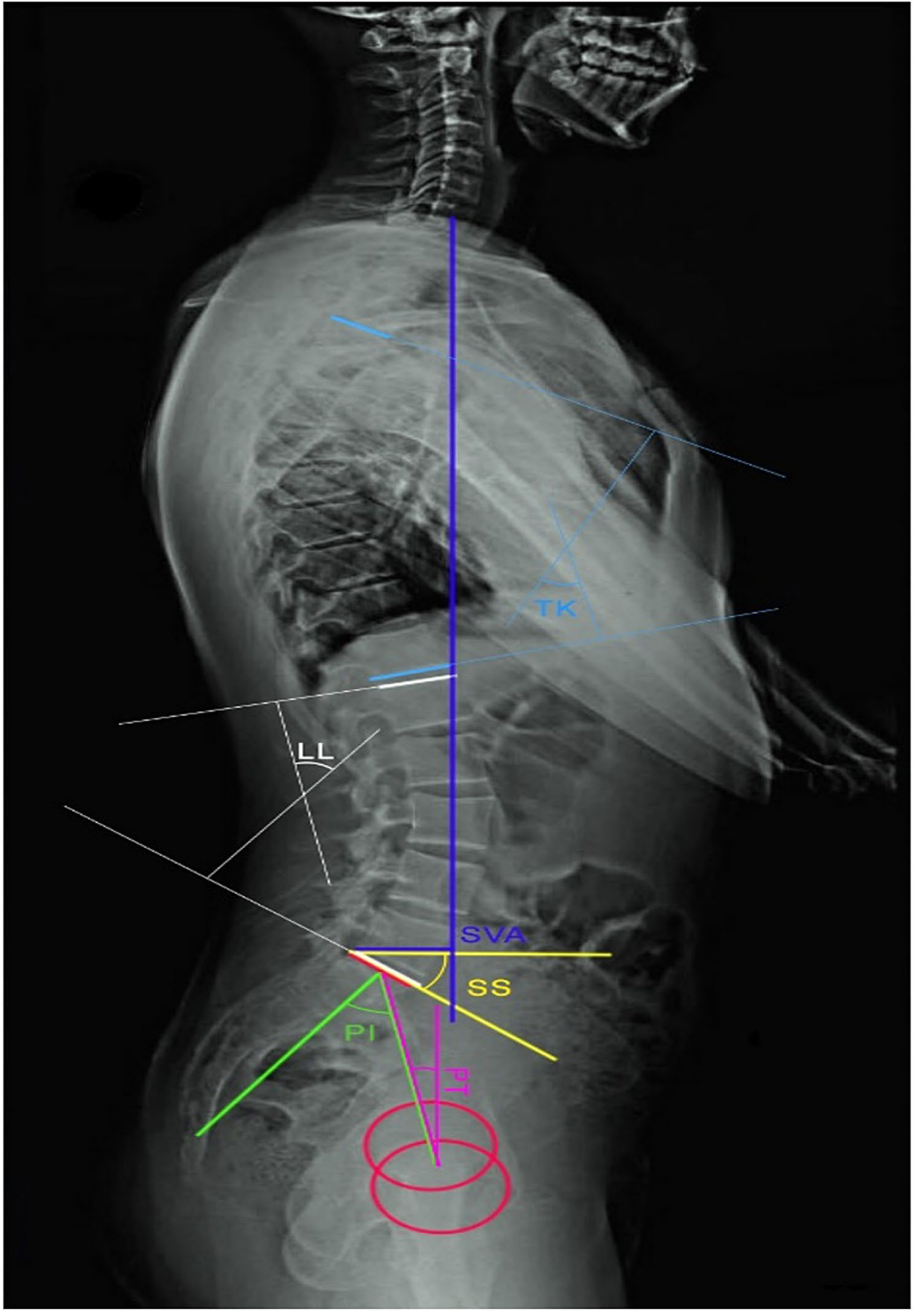
The measured spinopelvic indices

TK was measured as the Cobb angle between T4 and 12. LL was calculated as the Cobb angle between the superior endplate of L1 and the inferior endplate of L5 [21]. We measured PT as the angle between the line connecting the midpoint of the sacral endplate and the bicoxofemoral axis, and the plumb line [21]. SS was regarded as the angle between the tangent line on the sacral endplate and the horizontal line. PI was measured as the angle between the line perpendicular to the midpoint of the sacral endplate and the line connecting this point and the midpoint of the bicoxofemoral axis [21]. SVA was evaluated as the horizontal distance between the sagittal C7 plumb and the posterior superior corner of S1 [21] (Fig. 2).

To evaluate sagittal balance, a vertical reference line was drawn using the center of the body of C7. Then, the sagittal balance was defined based on its position relative to the posterior-superior edge of the S1. It was considered positive if it was in front of the point and negative if it was behind it, with normal sagittal balance in a normal person being negative.

### Statistical analysis

SPSS version 26 and G*Power version 3.1 software were used for the statistical analysis of data. The results for quantitative variables are expressed as the mean ± SD, and categorical variables are shown as the frequency and percentage. The Kolmogorov–Smirnov test of normality was used. To check the relationship between categorical variables, the chi-square test or Fisher’s exact test was used. The independent samples t-test or Mann–Whitney U test was used to compare quantitative variables between two types of imaging. The ICC with the two-way mixed model was used to evaluate reliability [19, 22]. ICC ≥ 0.9 was classified as excellent agreement, 0.7–0.9 as good agreement, 0.5–0.7 as moderate agreement, and ≤ 0.5 as poor agreement. Also, the coefficient of variation (COV) was evaluated to evaluate reliability. A P value ≤ 0.05 was considered significant. Bland–Altman plots were drawn for all parameters.

## Results

**Fig. 3:**
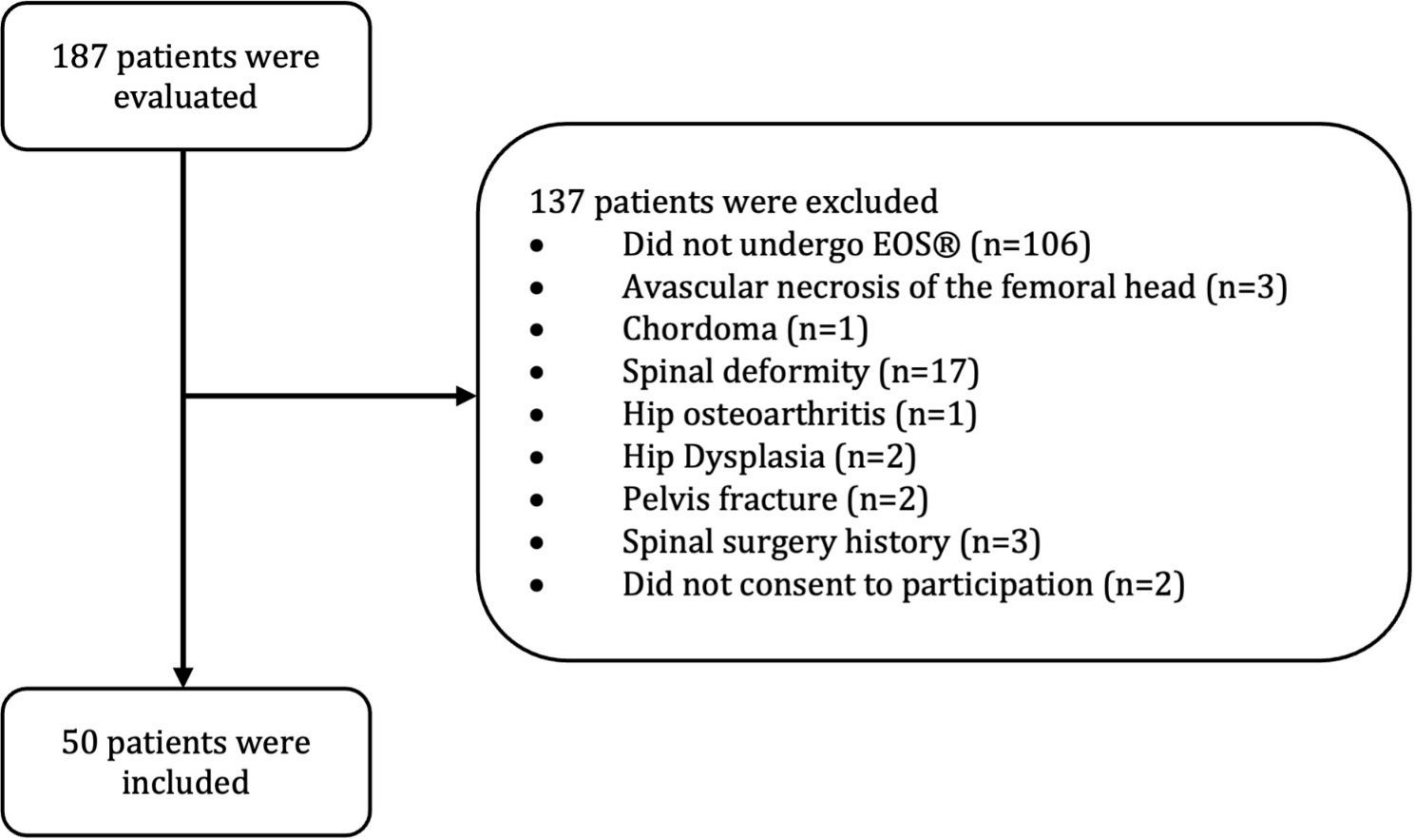
The study flowchart

A total of 50 consecutive patients were enrolled in this study. Figure 3 depicts the flowchart of the study inclusion. We performed a post-hoc power analysis, which showed a power of 95.00% with our sample size.

Overall, 30 patients (60%) were female. Table 1 demonstrates the demographics of the patients.

**Table 1:** Demographics of the patients

Variable	Mean	SD
Age	36.00	6.69
Height	164.60	8.89
Weight	80.34	9.73
BMI	29.86	4.47

Table 2 indicates the diagnoses, types of surgery, and past medical history among the patients. A total of 14 patients had a significant past medical history, among whom some patients had more than one condition, and 36 patients (72%) had unremarkable past medical history.

**Table 2:** Diagnosis, surgery type, and past medical history among the patients

Variable		Number	Percent
**Diagnosis**			
	Canal Stenosis	29	58
	Spondylolisthesis	12	24
	Disc Herniation	9	18
**Surgery Type**			
	Posterior Spinal Fusion	40	80
	Laminectomy	8	16
	Anterior Cervical Discectomy and Fusion	2	4
**Past Medical History**			
	Hypertension	11	22
	Diabetes Mellitus	6	12
	Ischemic Heart Disease	4	8
	Hypothyroidism	2	4
	Rheumatoid Arthritis	1	2

Table 3 shows the measurements of spinopelvic parameters by the two reviewers and the comparison of the measurements. There was no significant difference in the parameters measured using the conventional X-ray radiography and EOS® by the same reviewer. Additionally, the parameters of the same imaging system measured by different reviewers were not significantly different.

**Table 3:** Spinopelvic parameters as measured by the reviewers using conventional X-ray radiography and EOS®, and the comparison between them

Reviewer	Imaging	Parameter					
		**TK**	**LL**	**PT**	**SS**	**PI**	**SVA**
Radiologist	Conventional	31.07 ± 10.73	41.54 ± 16.13	16.90 ± 8.97	32.42 ± 10.13	49.18 ± 13.17	7.35 ± 10.09
	EOS®	29.32 ± 12.08	43.40 ± 16.22	15.44 ± 8.61	32.88 ± 9.28	48.08 ± 13.51	8.84 ± 9.29
	P value	0.448^1^	0.567^1^	0.409^1^	0.813^1^	0.681^1^	0.448^2^
Orthopedic Surgeon	Conventional	29.83 ± 11.71	42.85 ± 14.11	18.42 ± 9.55	32.04 ± 11.20	50.54 ± 14.38	6.94 ± 10.18
	EOS®	29.52 ± 12.43	41.56 ± 15.69	17.10 ± 10.39	32.99 ± 10.21	49.92 ± 14.36	8.98 ± 9.12
	P value	0.898^1^	0.714^1^	0.365^1^	0.659^1^	0.828^2^	0.302^2^
	P value^*^	0.584^1^	0.667^1^	0.360^1^	0.859^1^	0.627^2^	0.842^1^
	P value^†^	0.935^1^	0.607^1^	0.470^1^	0.955^1^	0.543^1^	0.855^2^

1: Independent samples t-test. 2: Mann Whitney U test

*: Comparison of conventional X-ray between reviewers

†: Comparison of EOS® between reviewers

Table 4 demonstrates the interobserver and intraobserver reliability of the parameters using ICC. As shown, the interobserver reliability was good and excellent for the conventional radiology system (0.871-980). Also, interobserver reliability for EOS® was excellent for all parameters (0.909–0.988). Moreover, both EOS® (0.986–0.998) and conventional radiology (0.971–0.999) systems had excellent agreement.

**Table 4:** Interobserver and intraobserver reliability evaluated by ICC

Reliability	Study	Parameter					
		**TK**	**LL**	**PT**	**SS**	**PI**	**SVA**
Interobserver	**Conventional**	0.923 (0.864–0.957)	0.871 (0.773–0.927)	0.947 (0.906–0.970)	0.885 (0.797–0.935)	0.935 (0.885–0.963)	0.980 (0.965–0.989)
	**EOS®**	0.956 (0.922–0.975)	0.952 (0.916–0.973)	0.921 (0.861–0.955)	0.909 (0.840–0.948)	0.940 (0.895–0.966)	0.988 (0.979–0.993)
Intraobserver	**Conventional**	0.992 (0.987–0.996)	0.989 (0.981–0.994)	0.971 (0.949–0.983)	0.997 (0.995–0.998)	0.990 (0.982–0.994)	0.999 (0.998–0.999)
	**EOS®**	0.996 (0.994–0.998)	0.996 (0.993–0.998)	0.995 (0.991–0.998)	0.986 (0.976–0.992)	0.998 (0.996–0.999)	0.996 (0.993–0.998)

Table 5 shows the interobserver and intraobserver reliability of the parameters using COV.

**Table 5:** Intraobserver and intraobserver reliability evaluated by COV

Reliability	Study	Parameter					
		**TK**	**LL**	**PT**	**SS**	**PI**	**SVA**
Interobserver	**Conventional**	21.3%	54.5%	45.4%	23.6%	14.4%	22.7%
	**EOS®**	36.6%	16.0%	35.2%	19.2%	14.9%	14.6%
Intraobserver	**Conventional**	9.3%	3.9%	18.6%	2.6%	6.6%	12.4%
	**EOS®**	8.3%	4.5%	10.9%	4.2%	2.2%	12.5%

**Fig. 4:**
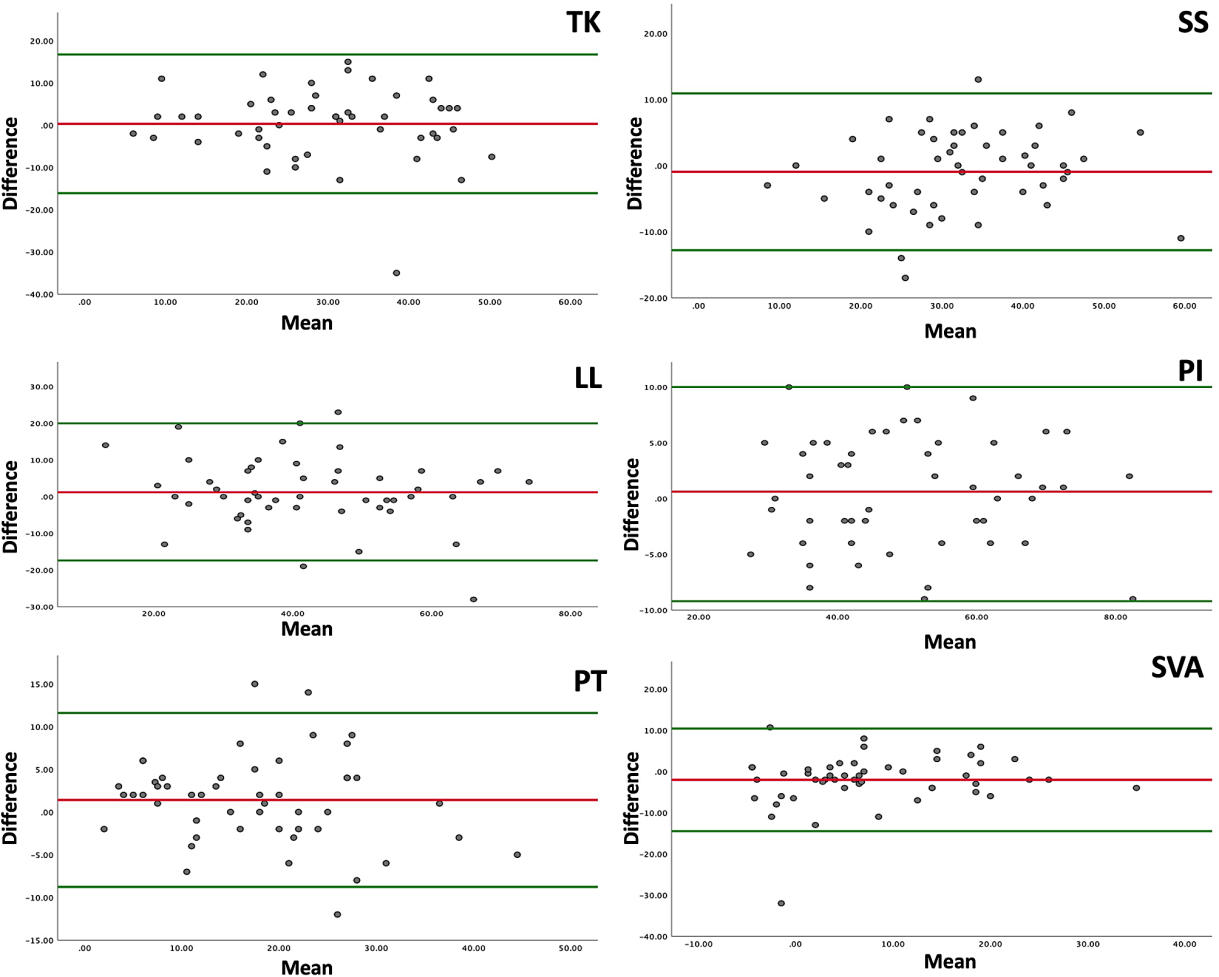
Bland-Altman plots for each parameter show good agreement between the two imaging methods

Moreover, Bland–Altman plots for each parameter showed good agreement between the two imaging methods (Fig. 4).

## Discussion

EOS® is a low-dose biplanar digital radiographic imaging system which obtains high-definition images with less irradiation and thus can avoid distortion [15–17]. It can also obtain 3D images other than 2D images [15]. In this study, we aimed to evaluate the reliability of spinopelvic parameters, including TK, LL, PT, SS, PI, and SVA, in EOS® and conventional X-ray C1S1 radiographs independently measured by two reviewers. We also compared the parameters measured by each reviewer between the two imaging studies and each parameter between the two reviewers. The results showed no difference in any parameter between EOS® and conventional X-ray C1S1 radiographs. Also, the parameters were not different between the two reviewers. The ICC was calculated to evaluate the interobserver reliability, which showed good and excellent agreement in the parameters; hence, it can be concluded that EOS® can be used instead of conventional X-ray C1S1 radiographs. Also, relatively low COV was observed evaluating intraobserver reliability; however, the interobserver reliability was higher, especially in the PT parameter in both imaging systems, which makes this parameter less conclusive. Interestingly, we found the LL parameter to show a much less COV in the EOS® system than in conventional X-rays. This may indicate the higher accuracy of EOS®.

EOS® depicts a full-body image in one shot without requiring stitches, while conventional X-rays need to stitch the images in order to show the entire spine [18]. Moreover, EOS® provides a comprehensive view, including the lower extremities. It helps determine a leg length discrepancy in adolescents and reveals compensatory changes in the sagittal plane due to degeneration, e.g., in the hip and knee in older patients [15, 23].

Wei et al. investigated the parameters of the lumbar spine and pelvis in EOS® whole-body and conventional X-ray imaging in 50 patients by two orthopedic specialists using Surgimap software. PI was significantly different between EOS® and X-ray imaging (P = 0.02). However, PT, SS, and LL were not significantly different. The researchers stated that the parameters of EOS® and X-ray imaging methods have a high similarity, and no difference in clinical guidance can be observed between these two methods [24]. Their results further prove our point that the EOS® system is as reliable as the conventional X-ray method to evaluate spinopelvic parameters. Fujita et al. evaluated the validity and reliability of spinopelvic parameters in upright CT scans against EOS® imaging in 26 adults with spinal deformity disorders, which were evaluated by two radiologists separately. Upright CT parameters showed high interobserver and intraobserver reliability in pelvic-spinal parameters, and full-body standing EOS® imaging had a higher error percentage compared to an upright CT scan [13]. This study also shows comparable findings in the spinopelvic parameters between the EOS® system and upright CT, and although a higher error percentage was observed with EOS®, it has much less radiation.

Wu et al. investigated the accuracy and validation of the parameters of the lumbar spine and pelvis in the EOS® whole-body imaging in 50 patients. Four main parameters, PI, PT, SS, and LL, were evaluated separately by two orthopedic specialists. The results showed that the difference between the angles in EOS® compared to the plain lateral radiograph of the lumbar spine was < 1˚, and a significant difference was observed between the two imaging modalities only in the PI parameter [20]. The results of Wu et al. are in line with our findings that the EOS® system provide images as reliable as conventional radiographs.

Lazennec et al. investigated the parameters in EOS® imaging of the lumbar-pelvic-femoral spine complex view in 46 patients (92 hips) and found that the angles in EOS® images allow the definition of intrinsic- and extrinsic-extension reserve to describe the mutual adaptive capacities of the hip angle with the lumbar spine [25]. Mac-Thiong et al. documented changes in sagittal parameters of the spine and pelvis during growth in 180 healthy 4- to 18-years old individuals. However, they did not report correlations between these parameters, although it is shown that the spinopelvic balance in adults strongly influences relationships between spine and pelvic geometry [26].

### Limitations

This study was not without limitations. First, patient posture may have changed during the performance of the two imaging modalities, for which we gave clear instructions to patients to assume the same posture for both studies, which was controlled by the thorough supervision of the radiology technologist. We believe future studies can explore the impact of patient posture changes on radiologic measurements. With strict control by the technician, we tried to minimize the effect of this variable, however, it can be explored not only in EOS® but in other modalities too. Additionally, we excluded patients with spinal deformities from the study; however, Chen et al. showed that spinopelvic parameters are the same in patients with or without spinal deformities [14]. We also used a single scanner for this study; thus, the generalizability of the study might be limited. Moreover, a larger sample size can result in more reliable findings.

In conclusion, EOS® imaging allows us to reliably evaluate spinopelvic parameters, including TK, LL, SS, PI, and SS, that are comparable to the measurements in conventional C1S1 lateral upright radiographs. This may help spine surgeons evaluate all neighboring joints with a single image and less exposure to the patient, and the pathologies which might have been missed would be noted.

## Data Availability

Research data is available from the corresponding author upon reasonable request.
